# Identification and Characterization of a Pepsin- and Chymotrypsin-Resistant Peptide in the α Subunit of the 11S Globulin Legumin from Common Bean (*Phaseolus vulgaris* L.)

**DOI:** 10.1021/acs.jafc.3c08744

**Published:** 2024-06-17

**Authors:** Liliana Santamaria, Aga Pajak, James D. House, Frédéric Marsolais

**Affiliations:** † Genomics and Biotechnology, London Research and Development Centre, Agriculture and Agri-Food Canada, 1391 Sandford Street, London, Ontario N5V 4T3, Canada; ‡ Department of Food and Human Nutritional Sciences, Faculty of Agricultural and Food Sciences, University of Manitoba, 204 Richardson Centre, 196 Innovation Drive, Winnipeg, Manitoba R3T 2N2, Canada

**Keywords:** legumin, 11S globulin, common bean, Phaseolus vulgaris, resistance to proteolytic digestion, pepsin, chymotrypsin

## Abstract

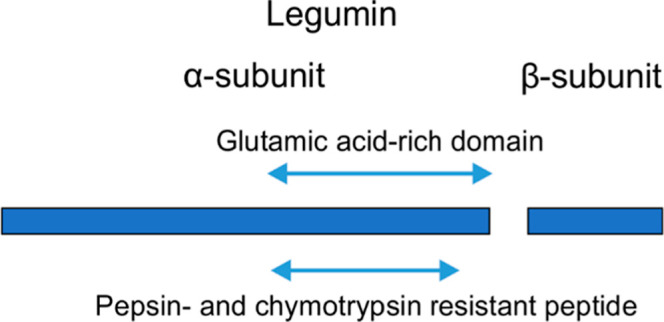

The 11S globulin legumin typically accounts for approximately 3% of the total protein in common beans (*Phaseolus vulgaris*). It was previously reported that a legumin peptide of approximately 20 kDa is resistant to pepsin digestion. Sequence prediction suggested that the pepsin-resistant peptide is located at the C-terminal end of the α-subunit, within a glutamic acid-rich domain, overlapping with a chymotrypsin-resistant peptide. Using purified legumin, the peptide of approximately 20 kDa was found to be resistant to pepsin digestion in a pH-dependent manner, and its location was determined by two-dimensional gel electrophoresis and LC-MS-MS. The location of the chymotrypsin-resistant peptide was confirmed by immunoblotting with peptide-specific polyclonal antibodies. The presence of a consensus site for proline hydroxylation and arabinosylation, the detection of hydroxyproline residues, purification by lectin affinity chromatography, and a difference in electrophoretic migration between the chymotrypsin- and pepsin-resistant peptides suggest the presence of a large *O*-glycan within these peptides.

## 1 Introduction

Pulse crops are predicted to play a central role in the global transition toward sustainable and healthy diets.^1,2^ Among them, common bean (*Phaseolus vulgaris* L.) constitutes the most important for human consumption, worldwide.^3^ Flour and protein concentrate from common bean and other pulses are incorporated as ingredients in food products, including meat analogues, using a variety of processing methods.^4,5^ Fractionation and processing can affect the digestibility of pulse protein.^6^ Digestibility is an important determinant of protein quality, which affects the availability of essential amino acids. Furthermore, stability to proteolytic digestion has been associated with the allergenicity of some food proteins.^7^ Resistance to digestive proteolysis means that an intact protein will increase its residence time in the gastrointestinal tract, where it may be more likely to stimulate an immune response.^8^

The major seed storage proteins of grain legumes include 7S and 11S globulins. In common bean, the 7S globulin phaseolin normally accounts for approximately 50% of the total seed protein, whereas the 11S globulin accumulates to approximately 3%.^9,10^ Legumin is relatively rich in sulfur amino acids compared with the 7S globulins. The legumin polypeptide precursor is cleaved into an acidic subunit (α) and a basic subunit (β), held by a disulfide bridge. This heterodimer assembles into a hexamer. Mühling et al. (1997) convincingly identified the legumin subunits from common bean through N-terminal sequencing.^10^ They reported that the major form of the α-subunit has a molecular mass of 54 kDa and the β-subunit has a unique size of 21 kDa. The results of purification and cDNA cloning revealed that the common bean 11S globulin belongs to a group of high molecular weight legumins with similarity to soybean glycinin A5A4B3 and peanut arachin 5.^11^ Momma (2006) reported that a 20 kDa peptide present in common bean paste was resistant to pepsin digestion, even after thermal processing.^12^ This peptide was tentatively identified as the β-subunit of legumin through N-terminal sequencing. The objective of this study was to further characterize the resistance of legumin to proteolytic digestion.

## 2 Materials and Methods

### 2.1 Sequence Analysis

Prediction of proteolysis resistant peptides was performed with ExPASy PeptideCutter tool.^13^ Either pepsin or chymotrypsin high specificity (C-term to [FYW], not before P) was specified as the enzyme. The deduced amino acid sequence was from GenBank accession no. ADR30064.1.^11^

### 2.2 Tissue Material

Seeds of the germplasm line of common bean (*P. vulgaris* L., Fabaceae) SMARC1N-PN1^14^ were harvested from the field in London, Canada. This line is an advantageous source of legumin as this protein is the most abundant in its seed, accounting for approximately 17% of the total seed protein.^15^

### 2.3 Purification of Legumin

Legumin was purified according to Yin et al.^11^ with modifications. Seeds were ground with a KLECO ball mill (Visalia, CA). After ammonium sulfate precipitation, the sample was desalted using a HiTrap Desalting 5 mL column (Cytiva, Marlborough, MA). Ion exchange was performed with a HiTrap Q FF 5 mL column. Size exclusion chromatography was performed with a HiLoad 16/600 Superdex 200 preparative grade using 50 mM Bis-Tris-HCl pH 6.5 and 150 mM NaCl as buffer. Alternatively, 0.6 g of flour was resuspended in 5 mL of lysis buffer (20 mM Tris-HCl, 150 mM NaCl), mixed using a mortar and pestle, and incubated at room temperature for 10 min. The homogenate was transferred to 1.5 mL tubes and centrifuged at 25,000*g* for 15 min. The supernatant was filtered through a 0.8/0.2 μm Acrodisc syringe filter (Pall, Mississauga, Canada). Five hundred microliters of peanut agglutinin agarose (Vector Laboratories, Burlington, Canada) were added to a Poly-Prep chromatography column (Bio-Rad Laboratories, Mississauga, Canada) and washed twice with Milli-Q water and four times with binding buffer (20 mM Tris-HCl, pH 7.4, 150 mM NaCl, 1 mM CaCl_2_, 1 mM MgCl_2_, and 1 mM MnCl_2_). The sample was diluted 1:1 with the binding buffer, loaded onto the column, and incubated at 4 °C for 15 min. The sample was passed through the column, and the column was washed four times with the binding buffer. The glycoprotein was eluted 5 times with 500 μL of glycoprotein elution solution (200 mM d-galactose in the binding buffer). Eluates were combined and desalted on a PD-10 column (Cytiva) in the appropriate buffer.

### 2.4 Pepsin Digestibility Assay

The test system is an in vitro digestion model using pepsin from porcine gastric mucosa, catalog no. P7012, >2500 units/mg protein (Sigma-Aldrich, Oakville, Canada), in simulated gastric fluid (SGF) at pH 2.0.^16^ SGF buffer was prepared by dissolving 70 mg of NaCl into 35 mL of ultrapure water and adjusting pH with 6 N HCl. Digestion was performed with 5 units of pepsin per μg of legumin. The assay was set up for a predetermined time course including 0, 1, 5, 10, 15, 30, and 60 min, by adding a fixed volume of the test protein, 12 μL of legumin at a concentration of 4 μg/μL, into 200 μL of SGF containing pepsin, and incubated at 37 °C. The reaction was terminated by adding 75 μL of sodium bicarbonate and 70 μL of 5 × SDS-PAGE sample buffer and heating at 75 °C for 5 min. Samples were analyzed by SDS-PAGE using a 10% polyacrylamide gel. Two-dimensional gel electrophoresis was performed using a Zoom IPGRunner System (Thermo Fisher Scientific, London, Canada) with a Zoom IPG Strip pH 3–10 Linear.

### 2.5 Chymotrypsin Digestion

α-Chymotrypsin from bovine pancreas, TLCK treated to inactivate residual trypsin activity, type VII, catalog no. C3142, ≥40 units/mg protein (Sigma-Aldrich), was resuspended in 2 mM CaCl_2_/1 mM HCl at 10 mg/mL. Legumin was digested with chymotrypsin at a ratio of 1:25 on a μg basis in 100 mM ammonium bicarbonate at pH 7.9 and 37 °C for 1 h. Legumin was digested at a concentration of 4 μg/μL, and a total of 48 μg was used per digest.

### 2.6 Mass Spectrometry

In-gel digestion was performed by using a MassPREP automated digester station (PerkinElmer, Guelph, Canada). Gel pieces were Coomassie destained using 50 mM ammonium bicarbonate and 50% acetonitrile, which was followed by protein reduction using 10 mM DTT, alkylation using 55 mM iodoacetamide, and tryptic digestion in 50 mM ammonium bicarbonate, pH 8. Peptides were extracted using a solution of 1% formic acid and 2% acetonitrile and lyophilized.

Prior to mass spectrometric analysis, dried peptide samples were redissolved in a 10% acetonitrile and 0.1% trifluoroacetic acid solution. MALDI matrix, α-cyano-4-hydroxycinnamic acid, was prepared as 5 mg/mL in 50% acetonitrile, 0.1% trifluoroacetic acid, and mixed with the sample at a 1:1 ratio (v/v).

Mass spectrometric data were obtained using an AB Sciex 5800 TOF/TOF system, MALDI TOF/TOF MS (Vaughn, Canada). Data acquisition and processing were respectively done using a TOF/TOF Series Explorer and Data Explorer (both from AB Sciex). The instrument was equipped with a 349 nm Nd:YLF OptiBeam On-Axis laser. The laser pulse rate was 400 Hz. Reflectron positive mode was used. Reflectron mode was externally calibrated at a 50 ppm mass tolerance and internally at 10 ppm. Each mass spectrum was collected as a sum of 500 shots. Data were searched with MS-Fit in ProteinProspector using UniProtKB Green Plants database (2020.09.02) using the following parameters: carbamidomethyl as fixed modification and oxidation (M) as variable modification; mass values: monoisotopic; peptide mass tolerance ±50 ppm; peptide charge state +1; and maximum number of missed cleavages of 3.

Alternatively, peptides were analyzed by LC-MS-MS. Peptides were separated using a nanoACQUITY UPLC System (Waters, Mississauga, Canada) on a 25 cm × 75 μm C18 reverse-phase column maintained at 35 °C. The flow rate was held at 300 nL min^–1^ throughout the run. All samples were trapped for 5 min at 99% water, 1% acetonitrile, and separated using a 5 to 40% gradient over 90 min, followed by a gradient to 95% acetonitrile over 5 min, and 10 min at 95% acetonitrile. Mass spectrometry was performed on an Orbitrap Elite (Thermo Fisher Scientific). The nanospray voltage was set at 2.7 kV, capillary temperature at 275 °C, and S-lens rf level at 54%. The full scan was acquired at an automatic gain control of 1 × 10^6^. Each sample was analyzed as a top 15, data-dependent acquisition experiment in the mass range of *m*/*z* 350–1600. Ions were isolated using an isolation width of 2.4 and then fragmented using high-energy collision-induced dissociation using a normalized collision energy of 35. High-energy collision-induced dissociation fragments were detected in the Orbitrap at a resolution of 30000. Data were searched with PEAKS 7.0 (Bioinformatics Solutions Inc., Waterloo, Canada), using a parent mass error tolerance of 20 ppm and fragment mass error tolerance of 0.8 Da. The maximum number of missed cleavages was equal to 3, with one nonspecific cleavage. Carbamidomethylation was selected as fixed modification, and all variable modifications. A maximum number of three variable post-translational modifications per peptide was allowed. The protein database searched was NCBInr. The LC-MS-MS data have been deposited to the ProteomeXchange Consortium via the PRIDE^17^ partner repository with the data set identifier PXD046332 and 10.6019/PXD046332.

### 2.7 Generation of Polyclonal Antibodies

Two polyclonal antibodies were raised against synthetic peptides present in the α-subunit of legumin: NH_2_-C^182^LAGNPDIEHPEAIK^195^-COOH, located upstream from the predicted protease resistant peptide, and NH_2_-^300^KEVEPLPHGK^310^C-COOH, located within the predicted pepsin-resistant peptide. Antibodies were produced by Biomatik (Kitchener, Canada).

### 2.8 Immunoblotting

Protein was separated on 10% SDS polyacrylamide gel and transferred to a PVDF membrane. The membranes were blocked in Odyssey blocking buffer (LI-COR Biosciences, Lincoln, NE) and PBS in a 1:1 ratio for 2 h. After blocking, membranes were incubated for 1.5 h with primary antibodies, washed three times with PBS buffer with 0.2% Tween-20 and subsequently incubated in secondary antibody for 1 h. IRDye 800 antirabbit secondary antibody (1:10000) was used for detection. The wet membranes were scanned on the Odyssey IR Imaging System in both 700 and 800 nm infrared fluorescent detection channels, giving rise to red and green channel detection.

## 3 Results

### 3.1 Sequence Analysis of Legumin

**Figure 1:**
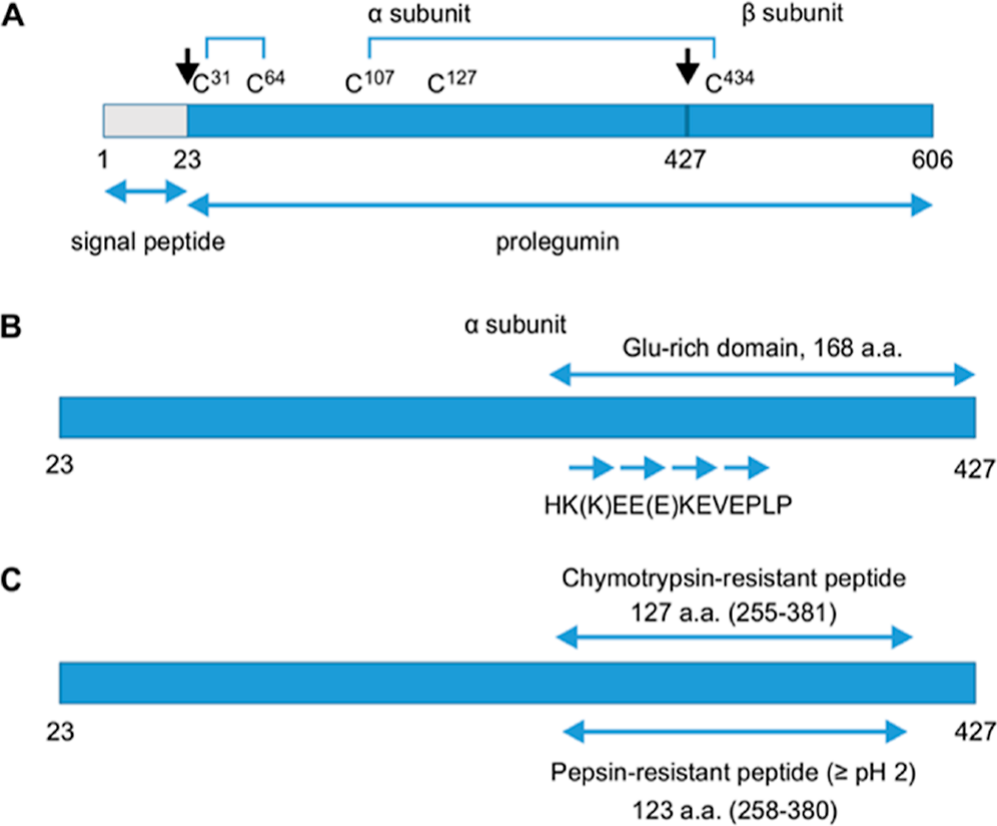
Schematic view of legumin and predicted protease-resistant peptides. (A) Cleavage of the signal peptide generates the prolegumin polypeptide, which is further cleaved into two subunits, α and β. There are five cysteine residues, two of which form an intramolecular disulfide bridge, with two other forming an intermolecular disulfide bridge between α- and β-subunits in the mature protein. (B) Close up view of the α-subunit. The C-terminal end contains a 168 amino acid glutamic acid-rich domain, characterized by the expansion of a motif repeated four times, whose consensus sequence is shown. (C) Position of the predicted chymotrypsin- and pepsin-resistant peptides (≥pH 2), which overlap with the glutamic-acid rich domain.

Legumin is a member of the 11S globulin family of proteins.^11^ It is a protein with 606 amino acid residues (Figure 1A). Cleavage of the signal peptide, before position 23, generates prolegumin. The mature legumin is processed into α-(acidic) and β-(basic) subunits via cleavage by an asparaginyl endopeptidase, also named legumain or vacuolar processing enzyme, before position 427.^10,15,18^ Mature legumin is predicted to have two disulfide bridges between Cys^31^ and Cys^64^ within the α-subunit and between Cys^107^ and Cys^434^ holding the α- and β-subunit together.^19^ An additional residue, Cys^127^, is not involved in a disulfide bridge. The α-subunit contains a glutamic acid-rich domain located at its C-terminal end, between positions 260 and 415 (Figure 1B). It is characterized by the expansion of a repeat, having the motif NH_2_-HK(K)EE(E)KEVEPLP-COOH that occurs four times within the sequence.^11^ The PeptideCutter algorithm predicted the presence of a 123 amino acid pepsin-resistant peptide, located within the glutamic acid-rich domain (positions 258–380), which overlaps with a predicted chymotrypsin-resistant peptide between residues 255 and 381 (Figure 1C).

### 3.2 Resistance to Pepsin Digestion

**Figure 2:**
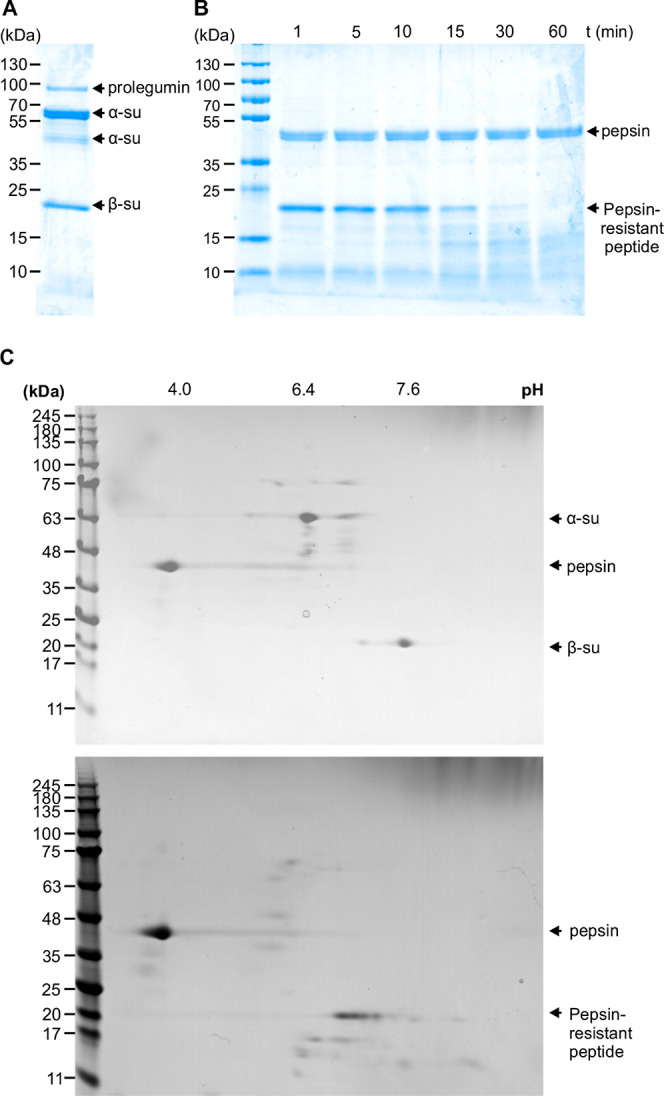
Characterization of the pepsin-resistant peptide. (A) SDS-PAGE of the purified legumin after ammonium sulfate precipitation, ion exchange, and size exclusion chromatography. (B) Time course of pepsin digestion of legumin. The pepsin-resistant peptide was identified after in-gel trypsin digestion and MALDI-MS. (C) Two-dimensional gel electrophoresis of purified legumin where the reaction with pepsin was quenched (top) and pepsin-digested sample (bottom). Protein spots were identified after in-gel trypsin digestion and LC-MS-MS.

**Figure 3:**
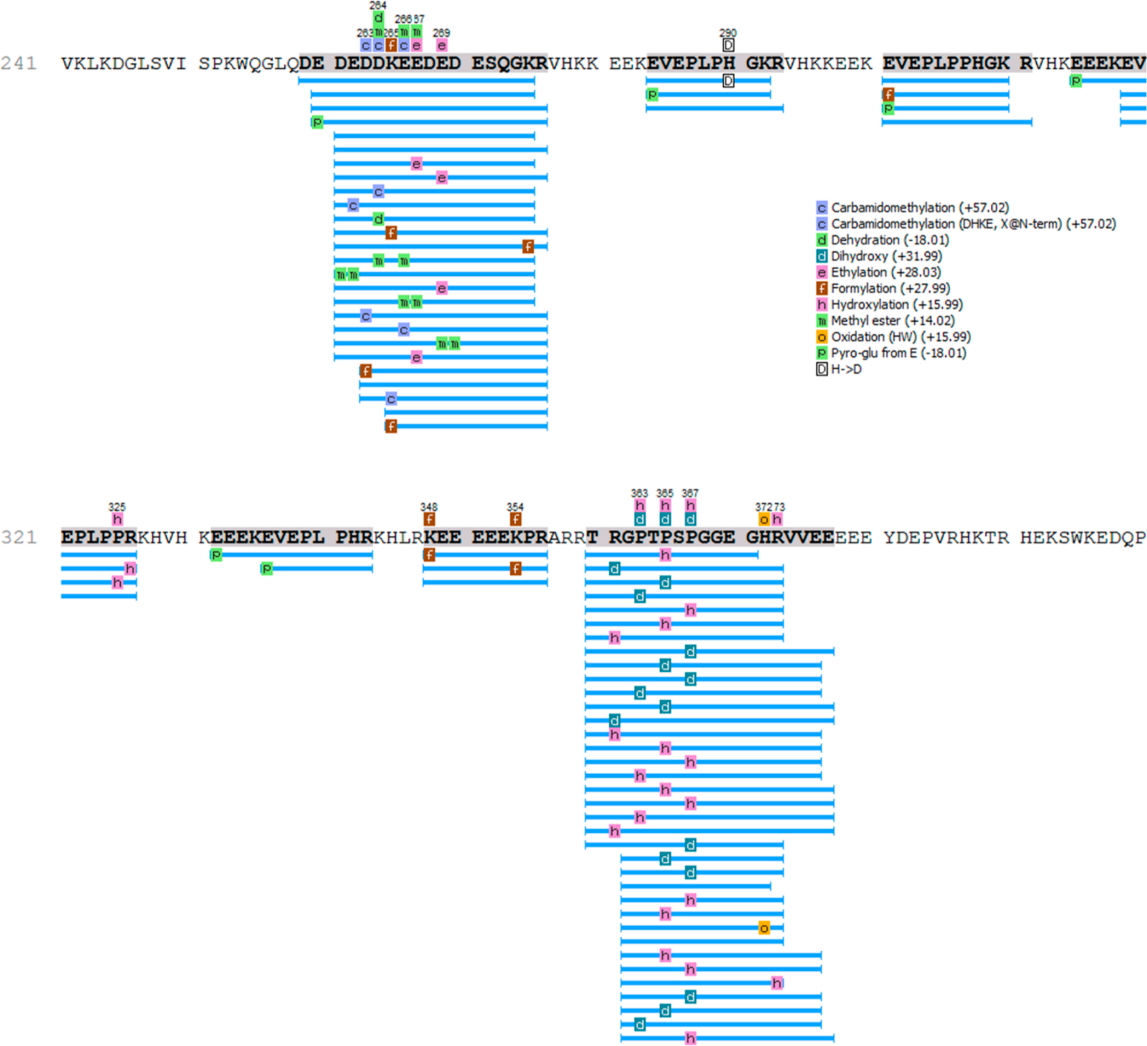
Proteomic analysis of the pepsin-resistant peptide. Coverage is shown of the predicted pepsin-resistant peptide sequence within the α-subunit of legumin by tryptic peptides derived from the 20 kDa pepsin-resistant peptide isolated from the two-dimensional gel spot in Figure 2C.

To evaluate the resistance of legumin to pepsin digestion, the protein was purified from mature seeds by a combination of ammonium sulfate precipitation, ion exchange, and size exclusion chromatography. As previously observed in SDS-PAGE, the purified legumin showed four distinct bands, corresponding to uncleaved prolegumin, a major α-subunit band of approximately 60 kDa, a minor α-subunit band of approximately 46 kDa, and β-subunit of approximately 22 kDa (Figure 2A).^11^ The purified legumin was subjected to pepsin digestion according to a protocol mimicking gastrointestinal digestion. Reaction products following a time course of digestion with pepsin were subjected to SDS-PAGE (Figure 2B). Within 1 min, bands corresponding to purified legumin disappeared, leaving two bands, one corresponding to pepsin (approximately 45 kDa) and a lower one (approximately 20 kDa). The levels of the 20 kDa band were progressively reduced over time, disappearing after 60 min. To confirm its identity, this band was excised, submitted to in-gel trypsin digestion and the resulting peptides were analyzed by peptide mass fingerprinting using MALDI-MS. The results confirmed the identity of this band as originating from legumin (Table 1 and Supporting Information). To characterize this in more detail, the purified legumin and digested sample were separated by two-dimensional gel electrophoresis (Figure 2C). The different protein spots were excised, digested with trypsin, and the resulting peptides were submitted to LC-MS-MS. A search tolerant of variable post-translational modifications was run to maximize coverage. Results confirmed the identification of each protein spot, including the 20 kDa pepsin-resistant peptide (Table 2). For this peptide, the majority of the resulting tryptic peptides covered the sequence of the predicted pepsin-resistant polypeptide (Figure 3). They mapped the N-terminal and C-terminal ends of the pepsin-resistant peptide as Asp^259^ and Glu^377^, respectively, which closely matches the sequence predicted by PeptideCutter. Only three of the peptides out of twenty eight were located outside of the predicted pepsin-resistant polypeptide, within the β-subunit (Table S1). The pepsin-resistant peptide overlaps with the glutamic acid-rich region, which includes the expansion repeat (Figure 1). The search identified several potential hydroxyproline residues, particularly within a glycine-, proline-, serine-, and threonine-containing motif, NH_2_-^360^TRGPTPSPGGE^370^-COOH, corresponding to a consensus site for proline hydroxylation and containing a predicted site for hydroxyproline arabinogalactosylation.^20^

**Table 1:** MS Fit Results for the 20 kDa Pepsin-Resistant Peptide Band in Figure 2B

MOWSE score	number of peptides	percent coverage	percent total ion chromatograms	mean error (ppm)	data tolerance (ppm)	accession	protein name
2.2 × 10^9^	20	37.1	64	–10.7	35.1	F8QXP7	legumin

**Table 2:** PEAKS Studio Results for the 20 kDa Pepsin-Resistant Peptide Spot in Figure 2C

accession	–10lg *P*	percent coverage	number of peptides	description
gi|312982406	153.79	21	28	legumin

### 3.3 Resistance to Chymotrypsin Digestion

**Figure 4:**
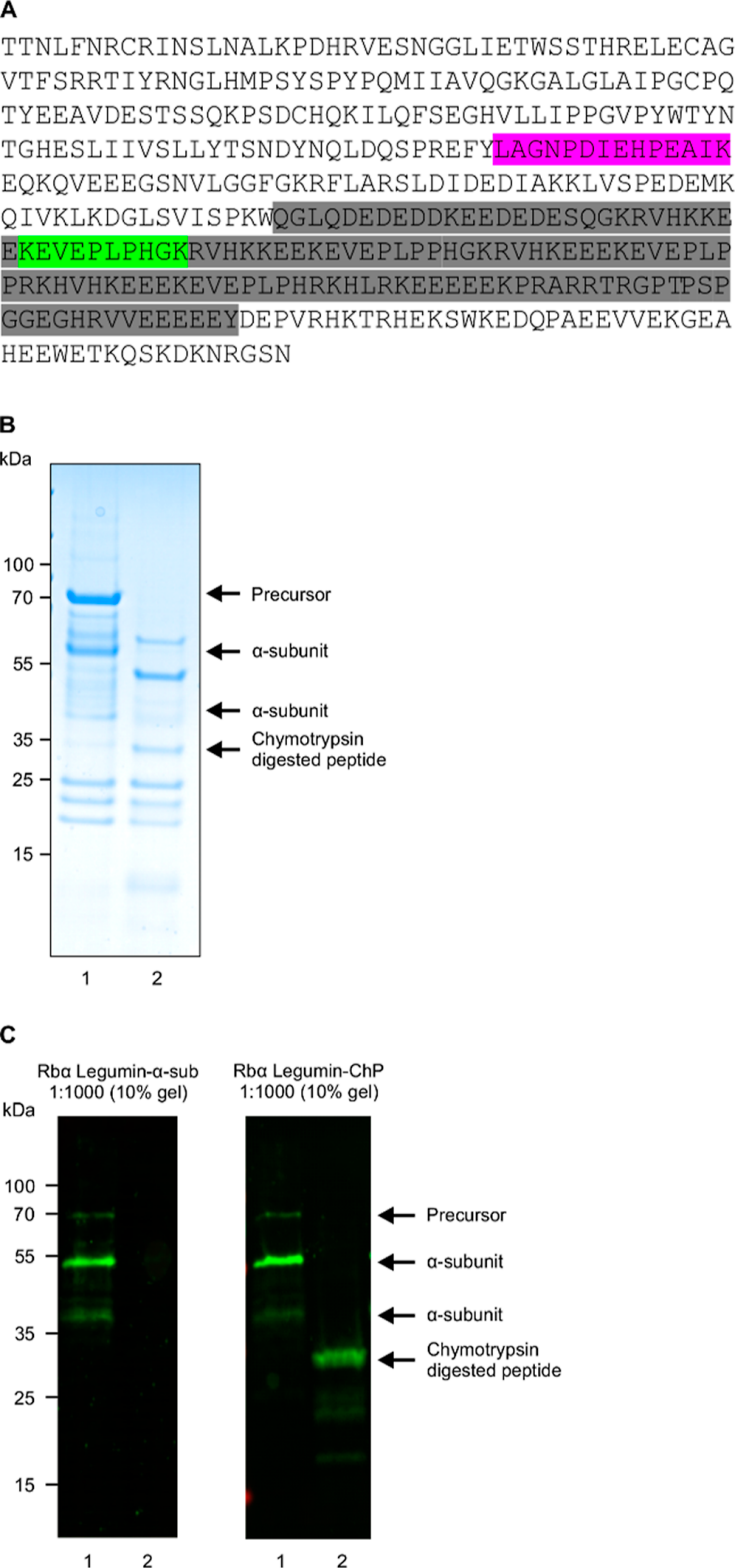
Chymotrypsin- and pepsin-resistant peptide overlap. (A) The sequence of the α-subunit is shown with the predicted chymotrypsin-resistant peptide highlighted in gray. Regions highlighted in violet and green represent peptides used to raise polyclonal antibodies. (B) SDS-PAGE of legumin purified by affinity chromatography on peanut agglutinin-agarose (lane 1) and after chymotrypsin digestion (lane 2). (C) Immunoblots of the SDS-PAGE in B with polyclonal antibodies directed against the α-subunit peptide located outside the predicted chymotrypsin-resistant peptide (left) or within (right).

An immunoblotting approach was used to investigate the localization of the chymotrypsin-resistant peptide. Polyclonal antibodies were raised against peptides present within the α-subunit, either outside or within the predicted chymotrypsin-resistant peptide (Figure 4A). For this experiment, legumin was purified by affinity chromatography with peanut agglutinin (Figure 4B). Antibodies raised against an α-subunit peptide located outside of the predicted chymotrypsin-resistant peptide reacted only with the purified legumin α-subunit prior to chymotrypsin digestion (Figure 4C). Antibodies raised against a peptide located within the predicted chymotrypsin-resistant peptide detected both purified legumin α-subunit, prior to chymotrypsin digestion, and an approximately 30 kDa band after chymotrypsin digestion, confirming that the sequences of the pepsin- and chymotrypsin-resistant peptides overlap.

## 4 Discussion

By using a combination of sequence prediction and experimental characterization, the pepsin- and chymotrypsin-resistant peptides were mapped to a segment of the α-subunit. However, the apparent molecular mass of the pepsin-resistant peptide was equal to 20 kDa (Figure 2C), higher than its predicted molecular mass of 14.1 kDa. It is very likely that the high percentage of glutamic acid influences the migration of this peptide. Guan et al.^21^ derived an equation whereby the percentage of glutamic acid and aspartic acid residues is used to estimate the difference between the observed and predicted migration of a polypeptide in SDS-PAGE. The pepsin-resistant peptide of legumin contains 26.9% of its residues as glutamic acid and 5% as aspartic acid. This should result in an increase in an apparent molecular mass of 6.7 kDa according to Guan’s equation, which corresponds closely to the difference observed.

Although the pepsin-resistant and chymotrypsin-resistant peptides overlap in sequence, a difference of approximately 10 kDa in apparent molecular mass was observed between them in SDS-PAGE (Figures 2B,C and 4C). A similar phenomenon was observed with the purified legumin, where the major form of the intact α-subunit has an apparent molecular mass approximately 10 kDa higher than expected, while a minor form migrates at the expected molecular mass (Figure 2A). It is likely that post-translational modification by glycosylation with a large *O*-glycan is responsible for the differences in migration. Under acidic conditions of pepsin digestion, glycans would be hydrolyzed, while they would be retained at neutral pH when used with chymotrypsin. This hypothesis is consistent with the presence of hydroxyproline residues and a sequence motif for proline hydroxylation and *O*-glycosylation in the pepsin-resistant peptide (Figure 3). Within this motif, Pro^363^ corresponds to a consensus site for efficient arabinogalactosylation, including elongation of the glycan side chain.^20^ This hypothesis is also supported by the affinity purification of legumin with peanut agglutinin, which binds the carbohydrate sequence Gal-β(1–3)-GalNAc (Figure 4B). Complex O-glycans, having a molecular mass of approximately 3–4 kDa, have been characterized in plants. A good example is in recombinant human interferon α2b produced in tobacco cells.^22^ The conclusion that the α-subunit of legumin may be glycosylated is unexpected as 11S globulins have been known to lack this post-translational modification.^9^

In this study, the identity of the pepsin-resistant peptide was clarified. The protein coverage (Figure 3) and immunoblotting data (Figure 4) unambiguously confirmed the sequence of the pepsin- and chymotrypsin-resistant peptide as corresponding to the glutamic acid-rich region of the α-subunit, as predicted by PeptideCutter (Figure 1). Resistance to digestion by these two enzymes is determined by their sequence specificity and the absence of key residues required for cleavage in the substrate.^23^ It is worth noting that the pepsin-resistant peptide and the β-subunit share similar molecular mass and pI (Figure 2C). Only a limited number of tryptic peptides were identified from the β-subunit, including one with a shortened N-terminal peptide lacking the Gly^428^ residue (Table S1); however, the majority covered the predicted pepsin-resistant peptide (Figure 3). In conclusion, the results from this study contribute to our knowledge of protein digestibility in common bean. This knowledge may be useful when analyzing the effects of different fractionation or processing methods.

## Supporting Information Available

MS-digest search results for MALDI-MS data (XLSX)
Peak lists for L20_1 and L20_2 replicates of the 20 kDa band and two negative controls from the same gel (TXT)
List of supporting peptides for identification of the pepsin-resistant peptide two-dimensional gel spot as legumin and their characteristics obtained from LC-MS-MS and PEAKS search (PDF)
