# Supplementary material for: Identification and Characterization of a Pepsin- and Chymotrypsin-Resistant Peptide in the α Subunit of the 11S Globulin Legumin from Common Bean (Phaseolus vulgaris L.)
Source: J Agric Food Chem. 2024 Jun 17;72(26):14844–50. doi: 10.1021/acs.jafc.3c08744 (PMC11228969; doi:10.1021/acs.jafc.3c08744)
Supplement: Supplementary file 3 — jf3c08744_si_003.pdf [file jf3c08744_si_003.pdf]

Supplementary Information

Identification and Characterization of a Pepsin and Chymotrypsin-Resistant Peptide in the α Subunit of the 11S Globulin Legumin from Common Bean (*Phaseolus vulgaris* L.)

Liliana Santamaria[a], Aga Pajak[a], James D. House[b] and Frédéric Marsolais[a]*

Table S1. List of supporting peptides for identification of the pepsin-resistant peptide two-dimensional gel spot in Fig. 2C as legumin (gi|312982406) and their characteristics obtained from LC-MS-MS and PEAKS search.

Peptide	Uniq	-10lgP	Mass	Length	ppm	m/z	z	RT	Scan	#Spec	Start	End	PTM
R.ASSADFFNPK.A	N	48.62	1082.5032	10	-1.3	542.2582	2	3.62	478	2	445	454	
K.E(-18.01)VEPLPHGK.R	N	44.25	986.5185	9	-0.5	494.2663	2	32.04	6809	10	284	292	Pyro-glu from E
K.E(-18.01)VEPLPHR.K	N	41.49	957.5032	8	-0.7	479.7585	2	33.46	7131	8	336	343	Pyro-glu from E
R.GPTP(+31.99)SPGGEGHRVVE.E	N	40.78	1506.7062	15	-1.2	754.3594	2	35.60	7673	3	362	376	Dihydroxy
R.TR(+15.99)GPTPSPGGEGHR.V	N	40.21	1420.6807	14	-0.7	474.5672	3	55.83	11976	10	360	373	Hydroxylation
R.GPTPSP(+15.99)GGEGHR.V	N	39.29	1163.5319	12	-1.1	388.8508	3	18.36	3262	53	362	373	Hydroxylation
E.DEDDKEEDEDESQGKR.V	Y	38.67	1922.7612	16	-0.4	641.9274	3	21.76	4213	84	261	276	
R.TRGPTP(+15.99)SPGGEGHRVVE.E	N	37.82	1747.8601	17	-0.6	583.6270	3	62.89	13260	25	360	376	Hydroxylation
R.TR(+15.99)GPTPSPGGEGHRVVEE.E	N	37.47	1876.9027	18	-1.3	626.6407	3	63.77	13413	3	360	377	Hydroxylation
R.TRGPTP(+15.99)SPGGEGHR.V	N	37.32	1420.6807	14	-0.9	474.5671	3	54.22	11702	10	360	373	Hydroxylation
E.DEDDKEEDEDESQGK.R	Y	37.22	1766.6602	15	-0.4	589.8937	3	13.42	2020	11	261	275	
R.TRGP(+15.99)TPSPGGEGHRVVE.E	N	35.52	1747.8601	17	-0.7	583.6269	3	63.87	13429	9	360	376	Hydroxylation
R.GPTPSP(+31.99)GGEGHR.V	N	35.01	1179.5269	12	-1.0	394.1825	3	16.31	2730	56	362	373	Dihydroxy
R.TR(+15.99)GPTPSPGGEGHRVVE.E	N	34.60	1747.8601	17	-1.6	583.6264	3	62.52	13195	15	360	376	Hydroxylation
R.TRGPTPSP(+15.99)GGEGHR.V	N	34.47	1420.6807	14	-1.5	474.5668	3	52.77	11482	4	360	373	Hydroxylation
E.DDKEEDEDESQGKR.V	N	33.86	1678.6918	14	-0.6	560.5709	3	20.14	3763	67	263	276	
R.GPTPSPGGEGHR.V	N	33.45	1147.5370	12	-0.4	383.5194	3	25.19	5110	20	362	373	
R.TRGPTP(+15.99)SPGGEGHRVVEE.E	N	33.10	1876.9027	18	-1.0	626.6409	3	64.56	13540	32	360	377	Hydroxylation
K.EVEPLPPR.K	N	32.85	935.5076	8	-0.6	468.7608	2	30.76	6491	30	319	326	
K.EVEPLPP(+15.99)R.K	N	32.71	951.5025	8	-0.7	476.7582	2	27.20	5619	6	319	326	Hydroxylation
R.TRGPTPSP(+15.99)GGEGHRVVEE.E	N	32.37	1876.9027	18	-0.7	626.6411	3	64.21	13484	12	360	377	Hydroxylation
R.GPTP(+15.99)SPGGEGHR.V	N	32.04	1163.5319	12	-0.6	388.8510	3	20.87	3967	10	362	373	Hydroxylation
K.E(-18.01)EEKEVEPLPPR.K	N	31.93	1432.7197	12	-0.3	717.3669	2	35.51	7650	14	315	326	Pyro-glu from E
K.EVEPLPPHGKR.V	N	31.68	1257.6829	11	-0.2	629.8486	2	50.97	11189	6	301	311	
K.E(-18.01)VEPLPPHGK.R	N	30.68	1083.5713	10	-1.0	542.7924	2	33.35	7104	9	301	310	Pyro-glu from E
R.GPTPSPGGEGH(+15.99)R.V	N	30.28	1163.5319	12	-1.0	582.7726	2	20.70	3922	3	362	373	Oxidation (HW)
R.GPTP(+15.99)SPGGEGHRVVE.E	N	30.10	1490.7113	15	1.2	746.3638	2	37.64	8228	4	362	376	Hydroxylation
R.GPTPSPGGEGH.R	N	29.45	991.4359	11	-0.2	496.7251	2	15.99	2645	2	362	372	
R.TRGPTP(+31.99)SPGGEGHRVVEE.E	N	29.40	1892.8976	18	-1.4	631.9722	3	60.34	12799	26	360	377	Dihydroxy
G.I(+57.02)EETLC(+57.02)TLK.L	N	28.82	1162.5903	9	-0.7	582.3021	2	3.67	494	1	429	437	Carbamidomethylation (DHKE, X@N-term); Carbamidomethylation
R.TRGPTPSP(+15.99)GGEGHRVVE.E	N	28.61	1747.8601	17	0.0	583.6273	3	62.14	13129	7	360	376	Hydroxylation
K.EVEPLPPR(+15.99).K	N	28.48	951.5025	8	-0.8	476.7581	2	27.01	5574	1	319	326	Hydroxylation
E.D(+14.02)E(+14.02)DDKEEDEDESQGK.R	Y	27.65	1794.6915	15	-0.1	599.2377	3	19.60	3610	1	261	275	Methyl ester
R.K(+27.99)EEEEEKPR.A	N	26.73	1200.5621	9	-0.7	401.1944	3	14.65	2305	15	348	356	Formylation
R.TRGPTPSP(+31.99)GGEGHRVVEE.E	N	26.63	1892.8976	18	-0.4	631.9729	3	60.89	12906	8	360	377	Dihydroxy

D.EDEDDKEEDEDESQGK.R	Y	26.38	1895.7028	16	-0.9	632.9077	3	13.85	2128	1	260	275	
E.DEDDKEEDE(+28.03)DESQGK.R	Y	26.09	1794.6915	15	-2.0	599.2366	3	20.30	3807	1	261	275	Ethylation
R.KEEEEEKPR.A	N	26.03	1172.5673	9	-0.4	391.8629	3	33.92	7243	39	348	356	
R.TRGPTP(+31.99)SPGGEGHR.V	N	25.98	1436.6757	14	-1.7	479.8983	3	50.65	11132	8	360	373	Dihydroxy
D.KEEDEDESQGKR.V	N	25.81	1448.6378	12	-0.3	483.8864	3	29.45	6165	27	265	276	
R.TRGP(+31.99)TPSPGGEGHRVVE.E	N	25.69	1763.8550	17	-1.4	588.9581	3	58.18	12387	1	360	376	Dihydroxy
R.GPTP(+31.99)SPGGEGHR.V	N	25.68	1179.5269	12	-0.9	394.1825	3	14.35	2237	13	362	373	Dihydroxy
R.GPTPSP(+31.99)GGEGHRVVE.E	N	25.41	1506.7062	15	-0.2	754.3602	2	35.40	7621	2	362	376	Dihydroxy
K.E(+27.99)VEPLPPHGK.R	N	25.36	1129.5768	10	0.3	565.7958	2	34.04	7270	2	301	310	Formylation
R.TR(+31.99)GPTPSPGGEGHR.V	N	25.23	1436.6757	14	-0.4	479.8990	3	50.92	11179	9	360	373	Dihydroxy
R.TRGPTP(+31.99)SPGGEGHRVVE.E	N	24.90	1763.8550	17	-1.2	588.9583	3	58.82	12502	17	360	376	Dihydroxy
K.EVEPLPPHGK.R	N	24.89	1101.5818	10	-0.7	551.7978	2	29.96	6295	6	301	310	
R.TRGP(+15.99)TPSPGGEGHRVVEE.E	N	24.18	1876.9027	18	-1.6	626.6405	3	63.68	13398	3	360	377	Hydroxylation
E.DEDDK(+27.99)EEDEDESQGKR.V	Y	24.17	1950.7562	16	-0.1	651.2593	3	16.84	2862	4	261	276	Formylation
E.DEDD(+14.02)KE(+14.02)EDEDESQGK.R	Y	23.76	1794.6915	15	-0.9	599.2372	3	20.78	3943	3	261	275	Methyl ester
K.EVEPLPHGKR.V	N	23.49	1160.6301	10	-2.5	581.3209	2	47.80	10583	7	284	293	
E.DEDD(+57.02)KEEDEDESQGK.R	Y	23.34	1823.6816	15	-1.4	608.9003	3	14.06	2172	1	261	275	Carbamidomethylation (DHKE, X@N-term)
R.GP(+31.99)TPSPGGEGHRVVE.E	N	23.04	1506.7062	15	-0.4	503.2425	3	35.03	7520	1	362	376	Dihydroxy
R.TRGPTPSP(+31.99)GGEGHRVVE.E	N	22.94	1763.8550	17	-0.2	588.9588	3	56.82	12161	2	360	376	Dihydroxy
E.DEDDKEE(+28.03)DEDESQGKR.V	Y	22.82	1950.7926	16	-2.0	651.2702	3	28.96	6041	1	261	276	Ethylation
E.DEDDKEEDE(+14.02)D(+14.02)ESQGKR.V	Y	22.73	1950.7926	16	-0.9	651.2709	3	28.30	5881	1	261	276	Methyl ester
K.E(-18.01)EEKEVEPLPHR.K	N	22.46	1472.7260	12	-0.6	491.9156	3	35.08	7533	2	332	343	Pyro-glu from E
R.TR(+31.99)GPTPSPGGEGHRVVEE.E	N	22.31	1892.8976	18	-0.5	631.9728	3	57.48	12268	1	360	377	Dihydroxy
E.DEDDKEEDEDESQGK(+27.99)R.V	Y	22.26	1950.7562	16	-0.8	651.2589	3	18.00	3163	4	261	276	Formylation
K.EVEPLPD(sub H)GK.R	N	22.25	982.4971	9	0.3	492.2560	2	28.03	5816	1	284	292	Mutation
R.TRGP(+31.99)TPSPGGEGHR.V	N	21.94	1436.6757	14	0.1	479.8992	3	48.60	10751	2	360	373	Dihydroxy
E.DEDDKE(+14.02)E(+14.02)DEDESQGK.R	Y	21.66	1794.6915	15	-0.9	599.2372	3	20.39	3833	1	261	275	Methyl ester
R.GPTPSP(+15.99)GGEGHRVVE.E	N	21.04	1490.7113	15	-0.2	746.3628	2	39.69	8764	4	362	376	Hydroxylation
R.KEEEEEK(+27.99)PR.A	N	20.40	1200.5621	9	-0.6	401.1944	3	19.04	3454	2	348	356	Formylation
R.VVNSEGIAVFDDELKK.G	N	20.24	1761.9148	16	-0.6	588.3118	3	53.82	11643	1	510	525	
E.D(+27.99)DKEEDEDESQGKR.V	N	20.17	1706.6866	14	-1.3	569.9021	3	15.78	2592	1	263	276	Formylation
D.EDEDDKEEDEDESQGKR.V	Y	20.07	2051.8040	17	-0.9	684.9413	3	22.33	4369	3	260	276	
D.E(-18.01)DEDDKEEDEDESQGKR.V	Y	19.97	2033.7933	17	-1.5	678.9374	3	18.61	3334	1	260	276	Pyro-glu from E
E.DEDDKEE(+28.03)DEDESQGK.R	Y	19.87	1794.6915	15	-0.8	599.2373	3	20.69	3918	1	261	275	Ethylation
Q.DEDEDDKEEDEDESQGK.R	Y	19.62	2010.7297	17	-1.4	671.2496	3	14.98	2385	1	259	275	
E.DEDDKEEDE(+28.03)DESQGKR.V	Y	19.37	1950.7926	16	0.2	651.2716	3	28.76	5992	2	261	276	Ethylation
E.DED(+57.02)DKEEDEDESQGKR.V	Y	19.23	1979.7827	16	0.2	660.9350	3	22.92	4522	1	261	276	Carbamidomethylation (DHKE, X@N-term)
R.TRGPTPSP(+31.99)GGEGHR.V	N	19.20	1436.6757	14	-0.9	479.8987	3	48.21	10673	1	360	373	Dihydroxy
E.DEDD(-18.01)KEEDEDESQGK.R	Y	19.17	1748.6497	15	-1.3	583.8897	3	14.94	2375	1	261	275	Dehydration
E.DEDDKE(+57.02)EDEDESQGKR.V	Y	18.99	1979.7827	16	-0.4	660.9346	3	24.51	4930	1	261	276	Carbamidomethylation (DHKE, X@N-term)
R.GPTPSPGGEGHR(+15.99).V	N	18.74	1163.5319	12	-0.7	582.7728	2	19.32	3532	1	362	373	Hydroxylation
D.K(+27.99)EEDEDESQGKR.V	N	18.61	1476.6328	12	-0.5	493.2180	3	14.73	2324	1	265	276	Formylation

E.DE(+57.02)DDKEEDEDESQGK.R	Y	18.18	1823.6816	15	-0.7	608.9008	3	14.15	2192	1	261	275	Carbamidomethylation (DHKE, X@N-term)
E.DDK(+57.02)EEDEDESQGKR.V	N	18.18	1735.7133	14	-0.8	579.5779	3	22.09	4302	1	263	276	Carbamidomethylation (DHKE, X@N-term)
R.GPTPSP(+15.99)GGEGHRVVEE.E	N	18.15	1619.7539	16	-0.4	540.9250	3	41.56	9227	1	362	377	Hydroxylation
R.TRGPTP(+15.99)SPGGEG.H	N	17.82	1127.5206	12	-0.5	564.7673	2	16.36	2743	1	360	371	Hydroxylation
